# Climate Change and Substance-Use Behaviors: A Risk-Pathways Framework

**DOI:** 10.1177/17456916221132739

**Published:** 2022-11-28

**Authors:** Francis Vergunst, Helen L. Berry, Kelton Minor, Nicholas Chadi

**Affiliations:** 1 Department of Special Needs Education, University of Oslo; 2 Department of Social and Preventive Medicine, University of Montreal; 3 Ste-Justine University Hospital Research Center, Montreal, Québec, Canada; 4 Australian Institute of Health Innovation, Macquarie University; 5 Center for Social Data Science, University of Copenhagen; 6 Data Science Institute, Columbia University; 7 Department of Pediatrics, Faculty of Medicine, University of Montreal

**Keywords:** addiction, substance abuse, mental health, developmental psychopathology, climate change, global warming, disasters, inequity, disease burden, long term

## Abstract

Climate change is undermining the mental and physical health of global populations, but the question of how it is affecting substance-use behaviors has not been systematically examined. In this narrative synthesis, we find that climate change could increase harmful substance use worldwide through at least five pathways: psychosocial stress arising from the destabilization of social, environmental, economic, and geopolitical support systems; increased rates of mental disorders; increased physical-health burden; incremental harmful changes to established behavior patterns; and worry about the dangers of unchecked climate change. These pathways could operate independently, additively, interactively, and cumulatively to increase substance-use vulnerability. Young people face disproportionate risks because of their high vulnerability to mental-health problems and substance-use disorders and greater number of life years ahead in which to be exposed to current and worsening climate change. We suggest that systems thinking and developmental life-course approaches provide practical frameworks for conceptualizing this relationship. Further conceptual, methodological, and empirical work is urgently needed to evaluate the nature and scope of this burden so that effective adaptive and preventive action can be taken.

Climate change is the most pressing public-health challenge facing humanity (World Health Organization [WHO], 2021). It is increasing the frequency and intensity of regional heatwaves, wildfires, droughts, floods, and storms (Beaumont et al., 2011; Intergovernmental Panel on Climate Change [IPCC], 2021), and without rapid emissions reductions, children born today will face up to seven times as many extreme weather events across their lives as did their grandparents’ generation (Thiery et al., 2021). The United Nations estimates that one half of the world’s 2.2 billion children are at “extremely high risk” from climate change because of harms to health, education, security, and access to essential services (UNICEF, 2021). Climate change’s impacts on human health are now extensively documented (Ebi et al., 2021; Hayes et al., 2018; Romanello et al., 2021; Sharpe & Davison, 2021), and more than 230 health-care journals recently published a joint editorial calling for urgent action to address the “catastrophic harm to health” (Atwoli et al., 2021). Despite these recognized impacts, the urgent question of how climate change may be affecting substance-use patterns has not been systematically addressed.

Substance-use disorders—which include the harmful use of tobacco, alcohol, recreational drugs (e.g., cannabis, cocaine, heroin, LSD), and prescription medications (e.g., opioids, benzodiazepines, barbiturates; McLellan, 2017)—account for 3.3% of the global disease burden and contribute to 16.8 million deaths each year (Degenhardt et al., 2018; Goodchild et al., 2018). They undermine economic and social participation and contribute to accidents, crime, financial precarity, increased mental- and physical-health morbidity, and the spread of infectious diseases, such as HIV (United Nations, 2012). The annual cost of substance-use disorders is approximately AU$136.9 billion in Australia (Government of Australia, 2021), £15.6 billion in the United Kingdom (Barber & Pratt, 2017), and more than US$750 billion in the United States (National Institute on Drug Abuse, 2020). In 2016, alcohol-use disorders were the most prevalent substance-use disorders worldwide, affecting approximately 100 million individuals, followed by cannabis- and opioid-use disorders, which affected 22.1 and 26.8 million individuals, respectively (Degenhardt et al., 2018). Only one in seven individuals with a substance-use disorder will receive treatment (United Nations, 2019), and relapse rates are approximately 40% to 60%, making them comparable to other chronic diseases (McLellan et al., 2000). Many more people use substances at harmful levels that do not reach clinical thresholds and are therefore omitted from official estimates (Substance Abuse and Mental Health Services Administration, 2020).

Substance-use disorders usually develop over time and are characterized by intense cravings, disruptions to work or social life, and inability to stop using despite a desire to change habits. The peak age of onset for substance-use disorders is 19.5 years, and, across the world, males are more likely to misuse substances than are females (Solmi et al., 2022). Risk factors for harmful substance use are complex and include genetic liability, personality factors, family and peer relationships, early substance-use exposure, mental disorders, poverty, stress, and the interplay between these factors (Ducci & Goldman, 2012; el-Guebaly et al., 2015; Morales et al., 2020). Climate change is now driving a wide range of complex, persistent, and interconnected stressors, from acute to chronic (Box 1), that are known to be associated with risky substance use and relapse vulnerability. Climate change is therefore likely to amplify patterns of harmful substance use worldwide, especially among young people and other vulnerable populations.

**Box 1.** Examples of Climate Change-Related Stressors and Impacts

• Hotter average summer temperatures
• More frequent and intense heatwaves, droughts, wildfires, storms, and floods
• Altered and lost landscapes, biodiversity, ecosystems, traditional lands, and valued places
• Water scarcity, food insecurity, famine, civil unrest, forced migration, war
• Increased range of insect-borne vectors (e.g., malaria, dengue), extended allergy season
(Intergovernmental Panel on Climate Change, 2021, 2022; Romanello et al., 2021)

As early as 2009, the American Psychological Association (APA) noted that substance-use disorders could be a by-product of increasing rates of mental-health disorders arising in the context of climate change (APA, 2009). Since then, a small number of studies have described patterns of harmful substance use in the context of severe weather events and postdisaster recovery and among indigenous communities responding to changes to landscapes, weather patterns, and traditional ways of living (Beaudoin, 2011; H. L. Berry et al., 2010; Bryant et al., 2014; Cepeda et al., 2010; Cunsolo Willox et al., 2013; Hansen et al., 2008; Hart et al., 2011; Lin et al., 2016; Lowe et al., 2017; Maclean et al., 2016; Milojevic et al., 2017; Peek-Asa et al., 2012; Peters et al., 2010; Rigby et al., 2011; Turner et al., 2013). Although recent reviews of climate change, mental health, and behavior (Clayton et al., 2021; Cruz et al., 2020; Evans, 2019; Middleton et al., 2020) have noted these effects, no study, to our knowledge, has explicitly explored the link between climate change and substance use at the broader conceptual level (Hwong et al., 2022). A search of PubMed, PsychInfo, and Google Scholar databases from January 1980 to October 2021 using the terms “climate change” and “substance use” and their synonyms (i.e., “climatic change” OR “global warming” AND “substance disorder” OR “substance abuse” OR “drug addiction” OR “drug abuse” OR “addiction” OR “addictive disorder”) returned no relevant results. This is concerning, both because of the already large global substance-use burden and because of the rapid pace of advancing climate change.

This article aims to address this conceptual knowledge gap in two principal ways. First, we examine plausible pathways and processes through which climate-change-related stressors could increase harmful substance use and relapse vulnerability. We do not attempt to specify all possible pathways but instead aim to describe the problem in broad terms to create the necessary foundation for further conceptual and empirical work. Second, because most substance-use disorders begin early in life, we describe two conceptual models that, when integrated, provide a promising framework for conceptualizing how climate-change–related stressors can increase substance-use vulnerability from the start of life onward. We conclude with a discussion of possible preventive and adaptive actions and outline research and measurement priorities going forward.

Throughout this article, we take an inclusive view of substance use that is not limited to formal substance disorders but includes use that is associated with harm or distress to individuals, households, communities, and society. We begin by considering the relationships among climate-related stressors broadly occurring in the contemporaneous space, then consider the association between climate change and specific risk pathways leading to harmful substance use, before expanding our framework to consider risks that occur across the life course. Because the dearth of literature on climate change and substance use precluded a systematic review, we conducted a narrative synthesis of the current literature on climate change, substance use, and mental health to investigate this relationship. Note that, although climate change has some benefits for certain populations (e.g., people living at high latitudes may benefit from increased agricultural yields and reduced risk from winter falls), the effects globally are understood to be overwhelmingly negative (Atwoli et al., 2021), and these consequently constitute the focus of our article.

## Pathways to Harmful Substance Use

**Fig. 1.**
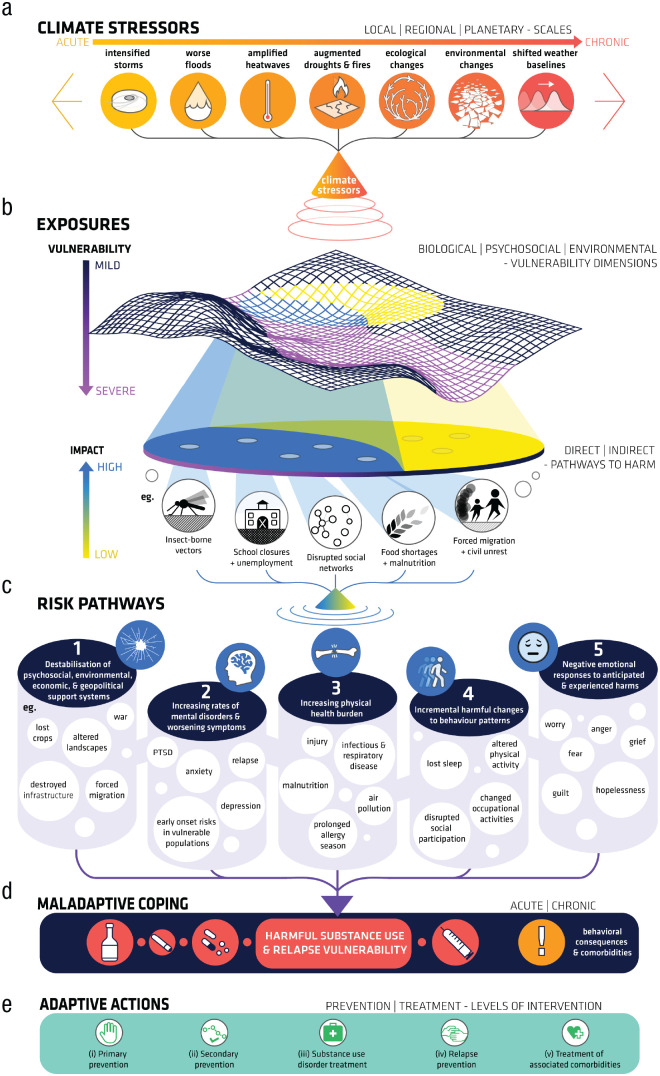
Schematic diagram of pathways and processes linking climate change to increased substance use. Climate change is increasing the frequency and severity of acute and chronic environmental stressors (a). The effect of these stressors varies as a function of individual vulnerability and the severity of exposure (b) and feeds into five independent pathways that increase the risk for harmful substance use (c). These pathways trigger maladaptive coping associated with increasing harmful substance use (d) requiring mitigative and adaptive action (e). Each of the five pathways could operate in isolation, in parallel, or interactively to increase risk. Although the five risk pathways are listed in the order that corresponds with their approximate importance in explaining the link between climatic stressors and harmful substance use, the actual variance explained by each will vary depending on individual and population-level vulnerability and resilience factors and the figure layout and proportions should be understood as illustrative only.

Human activities are altering the earth’s climate, and with it, the life-support systems on which human health and well-being depend. Average global surface temperatures have risen 1.1 °C since preindustrial times (IPCC, 2022). These changes are increasing the frequency and severity of environmental and human-caused disasters and disrupting many of the social and environmental determinants of good health, such as land- and sea-based livelihoods, social-support networks, education, health systems, physical infrastructure, valued landscapes, and socioecological relationships (Berrang-Ford et al., 2021; IPCC, 2021; Romanello et al., 2021; WHO, 2021). These unchecked and accelerating stressors—which disproportionately affect those who are already disadvantaged nationally and globally—could directly and indirectly increase harmful substance use and relapse vulnerability. This could occur through at least five pathways: psychosocial stress arising from the destabilization of social environmental, economic, and geopolitical support systems; increasing rates of mental disorders; increased physical-health burden; incremental harmful changes to established behavior patterns; and negative emotional responses to the anticipated and observed impacts of unchecked climate change (Fig. 1).

Figure 1 shows that climate change can directly and indirectly aggravate each of the five risk pathways, and each risk pathway can make a unique and independent contribution to harmful substance-use vulnerability. Furthermore, each pathway can operate with additive, interactive, and cumulative effects to increase the risk of substance use. The nature and extent of increased risk depends on factors such as the stressor and exposure types (e.g., acute vs. chronic, direct vs. indirect, low vs. high severity, isolated vs. compound) and individual vulnerability and protective factors. Although the distinction between multiple psychosocial pathways might appear contrived given that mental health and illness are increasingly understood to lie on a continuum rather than to belonging to qualitatively distinct categories (Martin et al., 2018), we argue that because these pathways *could* operate independently they should be treated separately (at least initially) so that their potential unique additive, interactive, and cumulative contribution(s) can be evaluated. Indeed, the operation of distinct pathways, both within and between individuals, could indicate distinct response approaches (e.g., prevention, early intervention), and they should therefore be conceptualized and evaluated separately.

1. Psychosocial stress arising from the destabilization of social environmental, economic, and geopolitical support systems

Climate change is already causing social and economic disruption by increasing the frequency, duration, and intensity of weather-related stressors. These may be acute (e.g., storms, wildfires, heatwaves), subacute (e.g., water scarcity), or chronic (e.g., rising sea levels, changing landscapes and ecosystems) events (IPCC, 2021; Romanello et al., 2021). Exposure to these events affects multiple aspects of functioning and daily living by contributing to job losses, school closures, homelessness, and forced migration (Berry et al., 2018; Horyniak et al., 2016); these are a major source of psychosocial distress and can activate acute or chronic stress responses in the body (Box 2). Extensive empirical evidence from human and animal studies indicates a robust association between stress and increased substance use and relapse vulnerability (Sinha, 2001, 2008). Numerous theories have been proposed to explain this association. Psychological models focus on substance use as a means of coping with stress through self-medication, whereas social epidemiology considers the role of social context and early exposure, and neurobiological explanations emphasize the role of sensitization of reward pathways that drive cravings and compulsive behaviors associated with addiction. The relationship between psychosocial and environmental stress and harmful substance use and relapse vulnerability has been comprehensively described in several reviews (Sinha, 2001, 2008).

**Box 2.** Climate-Change-Related Stressors and Harmful Substance Use

Example 1: Severe weather events
Current climate observations, models, and theories indicate that more frequent, intense, unpredictable, and long-lasting extreme weather events—such as storms, floods, heatwaves, and droughts—provide one of the most important region-specific pathways through which climate change will interact with underlying vulnerabilities to harm human health and well-being (Bell et al., 2018; Gamble et al., 2016). Epidemiological studies show a strong link between exposure to climate hazards such as hurricanes and wildfires and increased substance use (Beaudoin, 2011; Bryant et al., 2014; Lowe et al., 2017; Schiff & Fang, 2016). Young people are especially vulnerable, particularly when subject to repeated traumatic exposures, such as adolescents living in disaster-prone regions (Schiff & Fang, 2016).
Posttraumatic stress is the most common mental-health consequence in child survivors of extreme weather events (Barkin et al., 2021), with up to 71% of children experiencing symptoms postdisaster (La Greca et al., 2010; McDermott et al., 2014; Poulsen et al., 2015). Substance use and posttraumatic stress are functionally related to one another, but most studies indicate that posttraumatic stress precedes harmful substance use and relapse (Elman & Borsook, 2019; Jacobsen et al., 2001), suggesting a self-medication response (Alexander & Ward, 2018). Contrary to the notion that past stressors and traumatic events make people more resilient, longitudinal studies suggest that they may in fact increase vulnerability to future psychological distress and substance use (Fernandez et al., 2020; Khoury et al., 2010; McDermott et al., 2014).
The effect of acute and chronic weather-related stressors on harmful substance use are now so well recognized that the United States Substance Abuse and Mental Health Services Administration provides public information about the effects of weather-related disasters on mental health, including common psychological responses, anticipated symptoms, risk factors, and where to seek help (Substance Abuse and Mental Health Services Administration, 2020).
Example 2: Changing landscapes and ways of living
Climate change is increasing land and sea temperatures and thus altering landscapes, seascapes, and ecosystems on which people have survived for millennia. It is now clear that certain geographical areas are especially vulnerable to climate change (IPCC, 2021)—for example, the Arctic is heating up at roughly four times the rate of the global-warming average (Rantanen et al., 2022)—and the consequences of these changes on population mental health and substance-use vulnerability are documented in a growing literature.
Drawing on a series of in-depth interviews with community members and local and regional health professionals in the Canadian Artic, Cunsolo Willox and colleagues (2013) found that changes in weather patterns, snow and ice stability, and the extent of wildlife and vegetation patterns were associated with increased drug and alcohol use because of “disruptions in land-based activities and a loss of place-based solace and cultural identity” (p. 255). These patterns were amplified by previous traumas and mental-health stressors and were linked with increased risk of reported suicidal ideation. Similar negative psychological responses to changing landscapes and weather patterns have been reported among Aboriginal Australians (Rigby et al., 2011) and farmers with strong connections to the land (Polain et al., 2011).
Studies of this kind suggest that slow-moving damage to landscapes, ecosystems, place-based identity, and traditional ways of living—driven by climate change—constitute a new pathway to substance-use vulnerability. Further work is needed to map the complex interconnected relationships between the psychological states induced by these stressors and increased substance-use vulnerability using systems thinking so that the direction and strength of the putative pathways can be further validated.

2. Increasing rates of mental disorders

Mental disorders, including rates of psychiatric and neurodevelopmental disorders, are increasing as climate change advances (Amiri et al., 2021; Lawrance et al., 2021). This is because climate change is driving a suite of direct and indirect stressors—such as increased heat, humidity, rainfall, drought, wildfires, and floods—that are associated with psychological distress, increased hospital psychiatric admissions, self-harm and suicidal behaviors, and higher mortality rates among people with existing psychiatric disorders (Charlson et al., 2021; Nori-Sarma et al., 2022). Mental disorders and substance use are strongly linked (Kingston et al., 2017; van Duijvenbode & VanDerNagel, 2019). Epidemiological studies show that 50% of people with a substance-use disorder also have at least one mental disorder (Kelly & Daley, 2013; McCance-Katz 2020; Ross & Peselow, 2012), possibly as a result of common psychosocial and environmental risk pathways and shared genetic vulnerability related to specific neurotransmitter systems (Sharma & Morrow, 2016). Most people with substance-use and mental-health disorders report that the onset of their first mental-health disorder began before the onset of their first substance-use disorder (Kessler, 2004). This temporal pattern has been confirmed by prospective longitudinal studies and suggests that “self-medication” is a more likely response to deteriorating mental health for the majority of substance-use classes rather than substance use independently increasing the risk for mental disorders (Aas et al., 2021; Harrington et al., 2011). A review of Mendelian randomization studies indicates a bidirectional relationship for at least some mental-health disorders and substance-use classes, most notably with depression, bipolar disorder, and schizophrenia, which causally increase the odds of tobacco smoking, and vice versa (Treur et al., 2021). Taken together, current evidence indicates at least a partial causal relationship between mental disorders and harmful substance use and relapse vulnerability. This supports the notion that mental health is a relevant and indeed critical target pathway for mitigating the increasing rates of harmful substance use caused by climate change.

3. Increased physical-health burden

Harm to physical health is among the most extensively documented consequences of global climate change. It includes injury from extreme weather events; heat stress; extended allergy and asthma seasons; increased exposure to vector-borne diseases (e.g., malaria, dengue, Zika, Lyme); respiratory illnesses from air pollution; and malnutrition, dehydration, and developmental stunting stemming from reduced food and water availability and quality (Baker & Anttila-Hughes, 2020; Romanello et al., 2021; WHO, 2021). Harms that occur early in development undermine the attainment of subsequent developmental milestones and can have lifelong effects on disease vulnerability, including for noncommunicable disease risk (Barreca & Schaller, 2020; Frumkin & Haines, 2019; Heft-Neal et al., 2022; Mandy & Nyirenda, 2018; Olson & Metz, 2020). The growing physical-health burden associated with climate change could increase substance-use vulnerability through direct and indirect pathways. First, chronic health problems and disability are associated with higher rates of harmful substance use, possibly because of the stress of living with these disabilities (Hubbard et al., 1996; Wu et al., 2018). Climate-related increases in ill-health and disability worldwide could therefore correspondingly increase harmful substance use. Second, some common health problems require ongoing pain management. This increases population exposure to prescription medications with strong addictive potential (e.g., opioid-based analgesics; Dunn et al., 2010; Rosenblum et al., 2008), and, when use is protracted, increases the risk of development of a secondary substance-use disorder (Wilton et al., 2021). Third, physical-health problems interact with and increase mental-health vulnerability (Firth et al., 2019), creating an additional indirect pathway to substance-use vulnerability through mutually reinforcing cycles of harm to physical and mental health (Berry et al., 2018; Herrman & Jané-Llopis, 2012).

4. Incremental harmful changes to behavior patterns

The health effects of climate change do not only originate from weather extremes: The lived experience of shifting daily weather patterns can also precipitate changes in health behaviors that may be associated with substance use. For instance, evidence from natural experiments using large-scale data have shown that warmer-than-average temperatures in hot summer climates can amplify several behavioral outcomes also known to be linked to substance use: They can erode human sleep duration and quality (Minor et al., 2022; Mullins & White, 2019; Obradovich et al., 2017), increase injury risk (Parks et al., 2020), decrease learning and academic performance (Graff Zivin et al., 2020; Obradovich et al., 2018; Park, 2022), alter physical activity and active transport (Obradovich & Fowler, 2017; Obradovich & Rahwan, 2019), increase violent conflict (Miles-Novelo & Anderson, 2022), magnify depressive language (Burke et al., 2018), and depress emotional sentiment (Baylis et al., 2018; Noelke et al., 2016)—even when historically unusual temperatures are no longer socially remarkable (Moore et al., 2019). These daily behavioral changes may themselves initiate increased substance use or, possibly, follow from weather-associated increases in substance use or worsening of a preexisting mental disorder. For example, hotter weather could increase substance use directly by increasing alcohol consumption (Liu et al., 2020; Niu et al., 2020; Nori-Sarma et al., 2022; Schmeltz & Gamble, 2017; Wang et al., 2014; Yoo et al., 2021) or indirectly through mechanisms such as sleep loss (Cunsolo et al., 2020; Minor et al., 2022; Mullins & White, 2019; Obradovich et al., 2017). These, in turn, may increase the likelihood that people use alcohol or other depressants (e.g., benzodiazepines) to aid relaxation or stimulants to self-medicate next-day fatigue. Indeed, drug cue and craving indicators—such as the sight of drugs, paraphernalia, or contexts of use—play significant roles in drug use and relapse outcomes (Vafaie & Kober, 2022) and could help explain the link between harmful behavior changes and increasing rates of substance use. Innovative experimental designs are needed to assess the potential role and position of substance use in these putative causal pathways (see “Research Priorities” below).

5. Negative emotional responses to the observed and anticipated impacts of climate change

Population surveys from countries around the world show that climate change elicits strong and complex emotional responses, including feelings of worry, fear, anger, guilt, anxiety, grief, and hopelessness (Cunsolo et al., 2020; Dodds, 2021; Leiserowitz et al., 2020; Minor et al., 2019; for reviews, see Coffey et al., 2021; Ojala et al., 2021). The prevalence of reported negative climate emotions is already elevated internationally, especially among young people (Hickman et al., 2021). A survey of 10,000 people aged 16 to 25 across 10 countries found that more than 45% of respondents reported that their thoughts and feelings about climate change harmed their day-to-day life and functioning and that governments were failing to respond adequately, leaving young people with “no future” and feeling that “humanity [is] doomed” (Hickman et al., 2021). Although these negative emotional states should not be conflated with psychiatric diagnosis (Berry & Peel, 2015), damage to natural environments can lead to emotional distress (Albrecht et al., 2007), and emotional states affect substance use. For example, negative emotional states are associated with nicotine cravings among smokers (Heckman et al., 2013) and play an important role in substance-use relapse (Daughters et al., 2005; Strong et al., 2012). Sadness—but not all negative emotional states—has been linked with increased risk of long-term tobacco addiction (Dorison et al., 2020). More broadly, evidence from the psychiatric literature indicates a robust association between anxiety disorders and increased substance use (Smith & Book, 2008), although, as noted above, the direction of this association is uncertain (Treur et al., 2021), and generalizing from clinical diagnoses to subclinical emotional states requires further research. More work is also needed to assess the relationship between climate-related emotional responses and increased harmful substance use in both at-risk and population-based samples.

The pathways described above illustrate important and plausible links between climate-change-related stressors and harmful substance use, but they are not exhaustive, and many are not new. What is distinctive about these pathways in the context of climate change is the risks that feed into them, which are complex, interconnected, accelerating, and global in reach. They also emanate from a single environmental cause: rapidly rising average global temperatures as a result of increasing concentrations of greenhouse gasses in the atmosphere. Understanding these processes therefore requires a broad framework in which the stressors, exposures, vulnerabilities, and eventual impacts are considered as part of complex interconnected systems so that coherent mitigative and adaptive response strategies can be formulated. It is toward these conceptual challenges that we now turn.

## Conceptualizing Climate Change and Substance-Use Risks

Stress is arguably the most important general mechanism underlying the link between climate change and increased substance-use vulnerability. The term “stress” refers to processes involving perceiving, appraising, and responding to threatening, harmful, or challenging stimuli that activate emotional or physiological responses aimed at restoring homeostasis in the body (Lazarus, 2006). But “states of stress” are arguably too broad to helpfully conceptualize this relationship, and a key aim of this article is to specify key pathways and processes through which these effects may occur. One challenge, of course, is untangling the complex causal chains linking climate-change-related stressors and substance use; after all, substance-use disorders are already the result of complex, interacting chains of events that often begin early in life (even before birth) and extend across development (Tarter, 2002). Even a comprehensive cross-sectional analysis of contemporaneous risks—such as family dynamics, employment stability, and peer relationships—can be technically difficult. Consequently, we argue that, at this preliminary stage of the development of this field of research, risks are most helpfully conceptualized using a “systems thinking” approach.

Systems thinking is an inclusive approach for describing and analyzing a complex phenomenon with multiple interacting factors in a way that summarizes and simplifies the phenomenon and its key concepts. It focuses on how different parts of a “system” (a complex set of systematically and causally related factors) interact with and influence one another and which are nested within and interact with larger complex adaptive systems (Naylor et al., 2020). The approach has been successfully applied to understanding how climate change is related to mental health (Berry et al., 2018), and its utility and empirical validity was demonstrated in a recent systematic review (Hayward & Ayeb-Karlsson, 2021). It has also been incorporated into a developmental life-course approach, illustrating the ways in which climate change may harm psychosocial development through infancy, childhood, adolescence, and young adulthood (Vergunst & Berry, 2022). The main benefit of systems thinking is that, by elucidating the long and intricate causal chains linking prime mover events through initial vulnerabilities to eventual disorders, it provides a holistic understanding of the multiple, interacting exposures and pathways that combine to deliver an ultimate outcome. This, in turn, enables prevention and intervention efforts to be better targeted based on relevant individual characteristics and contextual factors, thus making them more likely to succeed (National Institute on Drug Abuse, 2018).

**Fig. 2.**
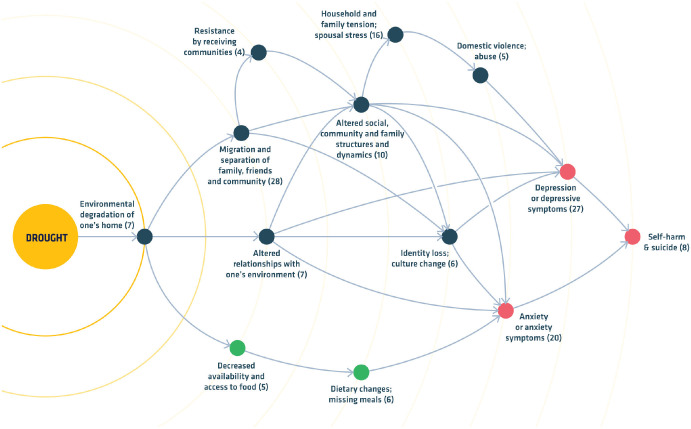
Effects of drought on mental-health outcomes as a result of the environmental degradation of one’s home, adapted from a systematic review (Vins et al., 2015). Arrows indicate the authors’ putative causal pathways; bracketed numbers indicate the quantity of articles meeting the search criteria for each risk factor. Note that feedback loops between risk factors (not illustrated) would further increase vulnerability. The figure draws on a “systems” approach to illustrate how a climate-change-related event—in this case drought—drives multiple interacting stressors that undermine mental-health resilience and could, through analogous stress processes, increase harmful substance use and relapse vulnerability. The example shows three of the five pathways described in the current article: psychosocial (blue), physical-health (green), and mental-health (red) stressors, each of which could independently and interactively increase substance use vulnerability.

Figure 2 illustrates how a systems approach can be applied to the mental-health context. It shows how a single climate-change-related stressor such as drought, which is becoming more frequent and severe in many regions with climate change, can harm mental health through the environmental degradation of one’s home (Vins et al., 2015), an example that is highly relevant to substance-use vulnerability (Sinha, 2008). The example emphasizes how a multidimensional weather stressor can influence complex multicausal outcomes, such as mental health, by triggering a chain of interconnected events that operate within broader systems to increase vulnerability. Although Figure 2 does not address substance use specifically, it illustrates how a systems approach can be used to conceptualize the relationship between climate-change-related stressors and substance-use vulnerability so that the pathways can be mapped and empirically evaluated (Hayward & Ayeb-Karlsson, 2021). The end benefit of this is to be able to locate precise timings and levels of intervention at specific places in the system, thus optimizing research designs, cost–benefit ratios, and treatment outcomes.

Although a systems approach is an important tool for understanding the complex interconnected pathways and mechanisms through which climate change is increasing risks for harmful substance use, current models do not specifically accommodate the complexity of life-course dynamics. For instance, prenatal and early life stressors, which are known to influence lifetime mental health and substance-use vulnerability (Khoury et al., 2010; Maclean et al., 2016), are not explicitly considered. Consequently, a systems approach can be enriched by integrating it with a developmental life-course perspective.

Developmental approaches are widely used in the mental-health and developmental sciences to understand the origins, course, and outcomes of mental disorders (Eme, 2017). The strength of developmental approaches is their consideration of multiple domains of analysis, including genetic, psychological, social, and environmental factors, and the dynamic interplay between them, both at a single point in time and across time. Furthermore, they emphasize the cascading nature of development, including how the timing, frequency, and severity of stressors can have lifelong effects on developmental outcomes (Beauchaine et al., 2018). Developmental approaches have been previously applied to understanding the pathways and processes that lead to substance-use vulnerability (Chassin et al., 2013; Glantz & Leshner, 2000; Maclean et al., 2016; Windle, 2010) and are especially relevant in the context of climate change, in which stressors are complex, interactive, and temporally distributed (Vergunst & Berry, 2022). The value of a developmental approach can be illustrated with examples drawn from the empirical literature.

Longitudinal studies show that stress exposure during the prenatal period can increase substance-use vulnerability in offspring decades later (Andersen, 2018). Furthermore, a wide variety of psychosocial and environmental stressors, including physical and sexual abuse, neglect, bereavement, family dissolution, and poverty, are associated with increased risk of substance-use initiation, maintenance, and dependence (Jordan & Andersen, 2017; Khoury et al., 2010; Morales et al., 2020). This is thought to occur partially through biological mechanisms whereby epigenetic processes cause early traumas to “get under the skin” and alter long-term physical- and mental-health disease liability (Aristizabal et al., 2019; Evans, 2016; Evans et al., 2012). As already noted, climate change will drive a wide range of exposures that increase the risk of often multiple and repeated acute, subacute, and chronic stressors across development (e.g., people living in weather-disaster-prone areas are more likely to be exposed to multiple disasters, with each event containing potentially multiple exposures). The earlier exposures occur in development, and the greater their number and severity, the greater their potential to “sensitize” the risk pathways that increase current and future substance-use vulnerability by operating with additive, interactive, and cumulative effects across time (Mandy & Nyirenda, 2018; Vergunst & Berry, 2022). A developmental approach further accommodates the fact that different risk pathways differentially affect substance-use vulnerability depending on the life-course stage. For example, young people are more likely to worry about their future, whereas older people experience greater physical-health burden, indicating distinct climate-related vulnerabilities and pathways for different subpopulations (Åström et al., 2019; Brown et al., 2006). Developmental approaches, particularly when situated within a broader systems-thinking framework, are therefore helpful for considering the long-term effects of climate change on substance-use vulnerability that cascade across the life course. They also contribute to developing a comprehensive conceptual framework for assessing risk and planning tailored intervention strategies at multiple levels, at different time points, and for different subpopulations (e.g., primary prevention for pregnant women, early intervention for adolescents in underserved communities, or treatment at the first onset of substance-use disorders).

## Measurement, Prevention, and Adaptation

Important progress has been made in the development and validation of measurement tools to track the health burden of climate change (see, e.g., the annual report of the *Lancet* Countdown: Tracking Progress on Health and Climate Change; Birkmann et al., 2022; Di Napoli et al., 2022; Romanello et al., 2021), but it focuses heavily on physical-health impacts. Much less is known about mental-health burden measurement, and substance use is barely mentioned (Berrang-Ford et al., 2021; Hwong et al., 2022; Romanello et al., 2021; WHO, 2022). Indeed, harmful substance use remains conspicuously absent from policy discussions and international climate-health monitoring efforts. Without this information, there is no clear roadmap to guide substance-use research, policymaking, adaptation, and prevention efforts as climate change advances. This lack of forward thinking increases the likelihood of focusing on treatments for clinical endpoints rather than on the (often distal) causal pathways that led to them, leaving us mopping up the floor instead of turning off the taps (Berry et al., 2018).

Our theory-driven, developmental approach aims to circumvent this problem. In addition to much needed conceptual work, we argue strongly for climate-change-informed tracking of the patterns and prevalence of harmful substance use in the coming decades, as well as a focus on robust empirical studies that test the a priori plausible pathways, processes, and mechanisms linking climate-change-related stressors with increased harmful substance use and relapse vulnerability (Box 3). This requires the deployment of robust measurement tools to track substance use arising from known and anticipated climate-change-related stressors (e.g., weather-related disasters, hotter average temperatures), investigating how climate-related harms to physical and mental health interact with and exacerbate harmful substance use, modeling the long-term global burden of climate-change-related harmful substance use, and identifying at-risk populations (including risk and protective factors) so that they can be monitored and supported. Birth cohort studies may be particularly valuable in addressing many of these questions because of their prospective, repeated-measures structure, phenotypic richness, and highly leverageable design that can include linkages with administrative data (e.g., medical, socioeconomic, and meteorological records) and across multiple generations (Vergunst & Berry, 2022). This work would be strengthened by establishing international interdisciplinary working groups focusing on climate change and substance use and on related and overlapping physical- and mental-health domains.

**Box 3.** Research Priorities

Conceptual development
• Develop and refine conceptual models that explain the interplay between climate change and harmful substance use (e.g., integrating systems thinking and developmental life-course approaches for specific sociodemographic subpopulations)
• Conduct risk-mapping exercises of climate-change-related stressors (Box 1) and substance-use vulnerability using a systems approach
• Use a developmental perspective to identify risk and protective factors at different points across the life course (e.g., adolescence, early adulthood, middle age, old age) for specific geographic and sociodemographic settings
• Refine and empirically test conceptual models that account for the relationship between substance use, mental health, and physical health
Monitoring and tracking
• Systematically review literature on climate-change-related stressors (e.g., extreme weather-related events) and their actual or potential effects on harmful substance use regionally and globally
• Track prevalence and patterns of substance use alongside documented climate-change-related stressors nationally, globally, and in high-risk populations in conjunction with existing reporting agencies such as the World Health Organization and *Lancet* Countdown (Romanello et al., 2021)
• Use longitudinal and intergenerational birth cohort studies to track the effects of climate-related stressors on substance use and mental and physical health across time, including the interaction between them (Vergunst & Berry, 2022)
Observational and experimental studies
• Conduct robust empirical studies that assess the effects of specific pathways (e.g., worry about climate change) and substance-use vulnerability while controlling for individual and location-specific time-varying confounding factors
• Examine the effects of different categories of stressors on substance-use type and patterns of use (e.g., substance-use hospital admissions in response to heatwaves vs. floods)
• Systematically investigate and isolate specific and general mechanisms underlying the association between climate-change-related stressors, increased substance-use vulnerability (e.g., general stress response, sleep loss), substance-use participation activities, and downstream health-related behavioral outcomes
• Examine the potential protective effects of climate-change education and participation in mitigation and adaptive actions as a means of reducing substance-use vulnerability among youth
• Leverage existing and emerging quasi-experimental research approaches and methods to assess the impact of climate-change-related stressors on harmful substance use, such as birth cohorts, multigenerational cohorts, and administrative and big-data approaches (Carleton & Hsiang, 2016)
• Test culturally appropriate prevention and support programs for populations at high risk from climate-change-related impacts, such as Indigenous people, those living in remote or agricultural communities, and people experiencing homelessness (Obradovich & Minor, 2022)

One in six people alive today are aged 15 to 24 years (United Nations, 2020), and the peak ages of onset for mental-health and substance-use disorders are 14.5 and 19.5 years, respectively (Solmi et al., 2022). This large and highly vulnerable population should form the backbone of prevention efforts and be targeted as early as possible, and preferably before the sensitive adolescent period begins (Jordan & Andersen, 2017). Efforts to prevent or delay initial substance-use exposure for as long as possible should also be a priority, in light of growing support for a causal relationship between tobacco and cannabis initiation and later problematic substance-use outcomes (Reed et al., 2022). Externalizing behavior problems are the most prevalent childhood psychiatric disorders, and they are more strongly associated with harmful substance use than with any other diagnostic category (Kessler, 2004), so young people exhibiting these problems will require particular monitoring and support. Further, people with preexisting substance-use disorders may be at disproportionate risk of heat-related morbidity and mortality (Oudin Åström et al., 2015; Page et al., 2012), and the effects of specific climate change-related stressors on health outcomes for this population should be investigated alongside resilience factors, such as adaptive coping (Fletcher & Sarkar, 2013; Rudzinski et al., 2017).

Other vulnerable populations, especially young people living on the frontline of climate change where extreme weather events are more common (e.g., inhabitants of Tuvalu who face inundation of their island home, Inuit communities that are losing traditional hunting grounds because of diminished sea ice; Cunsolo Willox et al., 2013; Minor et al., 2019), people experiencing homelessness (Kidd et al., 2021), those who depend on the land for their livelihoods (e.g., farmers, remote-dwelling Aboriginal communities), those living at the disadvantaged end of high health and income inequalities (Milojevic et al., 2017), and those living in rural and remote locations with limited capacity to respond and adapt should also receive monitoring and support. These efforts can inform health-care system preparedness and extreme weather-event-dependent public-health interventions to boost prevention and response planning for climate-change-related substance use and its downstream consequences (Parks et al., 2020). Furthermore, people living in low- and middle-income countries—especially in Africa and Central Asia—are disproportionately vulnerable to the harmful effects of climate change (Van Houtan et al., 2021) despite being among the least responsible for causing it, and their voices and expertise must be included in research and response planning (Godden et al., 2021). Such initiatives can bolster local adaptive capacity and climate literacy (Simpson et al., 2021) and increase the legitimacy of global-health response planning.

Climate change is already affecting every region on earth, and to the extent that we can no longer rely on mitigation strategies alone, aggressive adaptation is required to protect the health, well-being, and prosperity of future populations across the world (United Nations Environment Program, 2021). A recent review of the evidence on human adaptation to climate change across all sectors found that “adaptations were largely fragmented, local and incremental, with limited evidence of transformational adaptation and negligible evidence of risk reduction outcomes” (Berrang-Ford et al., 2021 p. 989). Within the health-care sector, nearly two thirds of countries worldwide do not have adequate national health-emergency frameworks and are unprepared to respond to climate-related health emergencies (Romanello et al., 2021). Mental-health services and support remain chronically underfunded worldwide: Although mental disorders affect around 1 billion people and cost the global economy more than US$1 trillion per year, they account for just 2.1% of national health-care expenditure (WHO, 2022). Substantial investments in health-care infrastructure and delivery will therefore be required to meet global substance-use prevention and treatment gaps in the coming years. At the individual level, strategies that offer win-win solutions by tackling climate change and improving mental health, well-being, and resilience should be encouraged, such as promoting active transport (e.g., walking/cycling instead of motor vehicles), dietary changes (e.g., more plant-based foods rather than animal products), and political and policy changes (e.g., taxing sugary/junk foods, “greening” cities and public-health services; Berry, 2009; Romanello et al., 2021; Vergunst et al., 2020).

Ultimately, solutions to the climate crisis lie in reducing greenhouse gas emissions as fast as possible, a fact that cannot be overstated and should be at the forefront of every discussion about climate change. Although further research is needed, the world—and, crucially, its leaders—already know enough to understand that translating this discussion into meaningful emissions reductions is all that now stands between humanity and a future that is, quite literally, terrifying.

### Implications

Our narrative review, with its proposed conceptual framework, advances knowledge in at least four ways: First, it reveals the understudied link between climate change and substance use and therefore the need for scientific inquiry in this area; second, it improves conceptual clarity about the pathways and processes through which these harms may occur and creates a foundation for further conceptual work and hypothesis generation; third, it links substance-use vulnerability with the published literature on climate-change harms to physical- and mental-health impacts and proposes the integration of existing conceptual frameworks (specifically systems thinking and a developmental life-course perspective) that emphasize the need for holistic long-term thinking and interdisciplinary collaboration to understand these relationships; and fourth, it provides an initial roadmap for academics, policymakers, clinicians, and advocacy groups to leverage and promote the development of new research and evidence-based policy responses and adaptation efforts.

## Conclusions

The aim of this article was to describe climate change as a risk factor for increasing rates of harmful substance use worldwide and to present a conceptual model for how these risks may operate and interact. We showed that a confluence of complex, interacting, and ongoing stressors is increasing substance-use vulnerability through multiple independent pathways across the life course. Risks begin before birth and cascade across development with additive, interactive, and cumulative effects. Because substance-use problems interact with and worsen physical and mental health, they will increase the overall global disease burden associated with climate change through negative reinforcing cycles. Further conceptual and methodological work is urgently needed to expand and refine our initial framework and to empirically test our proposed causal pathways with a view to informing research, adaptation, and prevention planning. Equally pressing is the need to increase awareness and engagement among politicians, community leaders, researchers, health professionals, and the public so that we are equipped to effectively respond to these emerging challenges.

## Transparency

*Action Editor:* Mirta Galesic

*Editor:* Klaus Fiedler

*Author Contributions*

F. Vergunst conceptualized the study and drafted the manuscript. K. Minor and F. Vergunst designed the figures with input from all authors. All authors contributed to the conceptual revision and editing of the manuscript and approved the final version for submission.
